# *Rickettsia africae* in the West Indies

**DOI:** 10.3201/eid1202.050903

**Published:** 2006-02

**Authors:** Patrick J. Kelly

**Affiliations:** * Ross University School of Veterinary Medicine, Basseterre, Saint Kitts and Nevis, West Indies

**Keywords:** *Rickettsia africae*, West Indies, Caribbean, African tick-bite fever

## Abstract

*Rickettsia africae* is the agent of African tick-bite fever, a mild but common disease of local persons and tourists in Africa. The major vector of this spotted fever group rickettsia is most likely *Amblyomma variegatum*, the tropical bont tick, which has become widely distributed through the West Indies in the last 30 years. This report reviews all available information on *R*. *africae* in the West Indies.

## Abstract 2

*Amblyomma variegatum* ticks should be eradicated to prevent *R. africae* and African tickborne fever from being established.

*Rickettsia africae* is a recently described spotted fever group (SFG) rickettsia that is the agent of African tick-bite fever (ATBF), a mild but common tickborne disease of local persons and tourists, in particular, in sub-Saharan Africa. The clinical and laboratory features of ATBF have recently been reviewed (*1*), as has the diagnosis of the disease (*2*). In Africa, the tropical bont tick, *Amblyomma variegatum*, is commonly infected with *R. africae* and is likely the major vector of the organism (*3*). This tick was introduced from Africa (Senegal) into the West Indies (Guadeloupe) in the early 1800s but has only spread widely and become endemic on many islands in the last 30 years (*4*). This spread was probably due to an increase in the between-island movement of livestock, major hosts of *A*. *variegatum* (*5*), and the introduction and spread of the cattle egret (*Bubulcus ibis*), a host of the immature stages of *A*. *variegatum* (*5*). Recent studies have demonstrated *R*. *africae* infections in *A*. *variegatum*, persons, and animals in the West Indies. In this report, the available information on *R*. *africae* in the region is reviewed.

## Epidemiology of *R*. *africae*

Early studies in southern Africa showed the bont tick, *A*. *hebraeum*, was commonly infected with *R*. *africae* (*6*). In feeding experiments, *R*. *africae* was maintained transtadially and transovarially in *A*. *hebraeum*, and the tick transmitted the organism at each feeding stage (*7*). Cattle and goats are common hosts of *A*. *hebraeum* and, when infected with *R*. *africae*, show no clinical or laboratory signs of disease. They are, however, intermittently rickettsemic and may then be sources of infection for ticks (*8**,**9*). While *A*. *hebraeum* is the most common vector of *R*. *africae* in southern Africa, epidemiologic evidence indicates that *A*. *variegatum* is the predominant vector in the rest of sub-Saharan Africa. This tick readily feeds on people (*10**,**11*) and is commonly infected with *R*. *africae* (16%–75%) in widely separated areas in Africa (*6**,**12**–**14*).

Although *R*. *africae* is widely distributed in Africa, and serosurveys have shown infections are extremely common in humans (up to 100%) (*1*), reports of ATBF in indigenous people are unexpectedly rare. This finding could be because they are generally infected at a young age, when the disease might be very mild or subclinical, and medical attention is not sought. Also, inoculation eschars are difficult to see in pigmented skin, and definitive diagnosis of ATBF requires sophisticated diagnostic tests not available in developing countries. The disease, however, is quite common in international travelers; up to 11% of visitors to disease-endemic areas have evidence of infection (*15**,**16*).

## *R*. *africae* in the West Indies

The first suspected cases of human spotted fever were reported from Guadeloupe in the 1960s (*1*). The patients had a history of tick bites and antibodies against SFG rickettsiae. Although rickettsiae were isolated from *A*. *variegatum* on the island, they were never definitively identified, and samples have been lost (*17*).

In 1998, Parola et al. (*18*) described a French woman who was bitten on the foot by a tick while visiting Guadeloupe. An erythematous nodule subsequently developed at the site as well as fever, elevated liver enzyme levels, and leukopenia. Serologic and adsorption studies suggested that she had been infected with *R*. *africae*. She recovered slowly when she was treated with doxycycline for 3 weeks. Subsequently, further human infections were documented on the island (*19*), and *R*. *africae* was detected in 27% of *A*. *variegatum* used for isolation experiments or polymerase chain reaction (PCR) analysis with *rOmpA* primers (*20*).

In 2002, Robinson et al. (*21*) used PCR with *rOmpA* and *gltA* primers to show that 84% of 75 *A*. *variegatum* collected from cattle in Antigua contained DNA of *R*. *africae*. In 2003, Kelly et al. (*22*) found 41% of *A*. *variegatum* from Saint Kitts and Nevis were positive for DNA of *R*. *africae* in PCRs in which *rOmpA* primers were used for the SFG rickettsiae. Positive ticks were found at 7 of 8 sites sampled, with prevalences varying from 14% to 71%. In the same year, Parola et al. (*23*) reported finding DNA of the *ompA* gene of *R*. *africae* in 7 (56%) of 12 *A*. *variegatum* tested from Martinique.

Although *Rhipicephalus* (*Boophilus*) *microplus*, the tropical/southern cattle tick, and *Rhipicephalus sanguineus*, the brown dog tick, are widespread in the Caribbean (*24*), they have not been implicated as vectors of *R*. *africae*. PCR with rO*mpA* primers of 52 *R*. *sanguineus* and 16 *R*. *microplus* from Saint Kitts and Nevis did not show DNA of SFG rickettsiae (unpub. data). Similarly, SFG rickettsial DNA was not identified in 6 *R*. *microplus* and 11 *R*. *sanguineus* from Martinique (*23*) or in 6 *R*. *microplus* from Antigua (*21*).

The studies described show that *A*. *variegatum* is commonly infected with *R*. *africae* in the West Indies. In the only published serosurvey conducted in the region (*20*), high prevalences of antibodies to *R*. *africae* were found in Guadeloupean cattle (81%) and goats (87%), which are common hosts of *A*. *variegatum* (*24*). Antibodies to *R*. *africae* were also highly prevalent (49%) in local people from Guadeloupe. The prevalence in men was significantly higher than in women, possibly because men were more likely to be exposed while working outdoors. The West Indian population, then, appears to be commonly exposed to *A. variegatum* that transmits *R*. *africae*. As is the case in Africa, however, clinical cases of ATBF in local persons are unexpectedly rare; the only reported cases of ATBF contracted in the region have been in tourists (*18**,**19*).

Recently, programs have been introduced to eradicate *A*. *variegatum* from the Caribbean (*25**–**27*). The principal justification for the projects has been the economic loses to island economies caused by animal diseases associated with *A*. *variegatum*, mainly heartwater and dermatophilosis. Also of great concern have been the huge economic loses that would be anticipated if the tick and its animal diseases were introduced into South, Central, and North America (*28**,**29*). The programs have met with mixed success, and although some islands have been certified provisionally free of the tick, others remain infested or have reinfestations or recrudescences of *A*. *variegatum*. No attempts appear to have been made to control the populations of cattle egrets, which are hosts of *A*. *variegatum* and can migrate long distances, even as far as the Florida Keys (*30*).

## Conclusions

Until *A. variegatum* is eradicated from the West Indies, local health workers and those treating persons who have traveled to the area should suspect ATBF in patients who seek treatment with a history of tick bites and clinical signs of fever, headache, and multiple eschars. Further, vigilance is required to prevent transportation of *A*. *variegatum* or rickettsemic animals to the mainlands of North, South, and Central America because this importation might enable *R*. *africae* and ATBF to become established in these areas. The potential impact of *R*. *africae* on the health of indigenous people and tourists in the West Indies and its potential introduction into the Americas further justifies the eradication of *A*. *variegatum* from the region.
